# Mobility Network Analysis of the Poultry Sector in Morocco: A Tool for the Surveillance and Prevention of Highly Pathogenic Avian Influenza

**DOI:** 10.3390/vetsci13090858

**Published:** 2026-08-24

**Authors:** Fadoua Boudouma, Yahya Farhi, Mohamed Dehhaoui, Hicham Hajji, Oumayma Arbani, Kenza Aitelkadi, Siham Fellahi

**Affiliations:** 1 Department of Animal Health Regulation, National Office of Food Safety (ONSSA), Rabat 10000, Morocco; 2 Department of Veterinary Pathology and Public Health, Institut Agronomique et Vétérinaire Hassan II, Rabat 10000, Morocco; yahyafarhi.vet@gmail.com (Y.F.); o.arbani@iav.ac.ma (O.A.); s.fellahi@iav.ac.ma (S.F.); 3 Department of Applied Statistics and Computer Science, Institut Agronomique et Vétérinaire Hassan II, Rabat 10000, Morocco; m.dehhaoui@iav.ac.ma; 4 Department of Cartography-Photogrammetry, Institut Agronomique et Vétérinaire Hassan II, Rabat 10000, Morocco; h.hajji@iav.ac.ma (H.H.); k.aitelkadi@iav.ac.ma (K.A.)

**Keywords:** social network analysis, mobility, poultry sector, HPAI, Morocco

## Abstract

Background: Highly pathogenic avian influenza (HPAI) poses a critical global threat to both the poultry industry and public health. Although Morocco currently maintains HPAI-free status, the country faces substantial risk due to its location along major migratory flyways and its extensive commercial trade networks within the poultry sector. Available data indicate that the links between live bird markets, production farms, and related facilities in the poultry sector constitute a pivotal determinant of disease epidemiology. This study aimed to analyze these movements and determine how they can influence the spread of the disease and to guide policymakers in developing effective risk-based surveillance and control strategies tailored to the local context.: A questionnaire-based cross-sectional survey was conducted across the Casablanca-Settat region, including nine provinces and 138 municipalities, to investigate the movement patterns within the poultry sector in this region. Social network analysis (SNA) was employed to construct a movement network, and findings were spatially visualized using Geographic Information Systems (GIS) and analyzed through network analysis in R.: A total of 945 transport routes were recorded in 126 municipalities. Centrality measures identified three predominant network nodes exhibiting high degree and betweenness centrality values. Our findings indicate that although the network has a low density, the transmission of IAHP remains critical due to frequent bidirectional links and a heterogeneous network structure. The high concentration of movements by a few “hub” municipalities leads to an early emergence and rapid dissemination.: The identification of highly influential nodes allows veterinary authorities to prioritize and target surveillance activities toward municipalities with the greatest likelihood of disease introduction or persistence. The network’s structural connectivity suggests a theoretical potential for rapid HPAI spread, underscoring the importance of the frequent bidirectional links identified between municipalities. This protects both Morocco’s poultry industry and public health.

## Simple Summary

This study analyzed movement patterns between live bird markets, poultry farms, and related facilities across the Casablanca-Settat region of Morocco, using network analysis to understand how the structure of these connections could influence the spread of highly pathogenic avian influenza (HPAI) within the national poultry sector. The results revealed that poultry movements are highly concentrated around a small number of key municipalities, known as “hubs,” which play a central role in connecting the network. This uneven distribution means that a disease introduction at one of these hubs could trigger rapid and widespread dissemination throughout the region. By identifying the most influential nodes in the network, this study provides veterinary authorities with a practical tool to prioritize surveillance and control efforts of the municipalities most likely to introduce or sustain disease. Targeted interventions at these critical points, rather than blanket measures across the entire network, would significantly improve the efficiency of HPAI prevention and response. These findings contribute to safeguarding both Morocco’s poultry industry and public health, and lay the groundwork for a more evidence-based national strategy for avian influenza surveillance and control.

## 1. Introduction

Highly pathogenic avian influenza (HPAI) constitutes a major and persistent threat to both the poultry industry and global public health. Characterized by rapid spread and high mortality rates in poultry, the virus leads to severe sanitary and economic losses within affected production systems. Over recent decades, HPAI has caused numerous epizootics across multiple regions worldwide, resulting in the loss of millions of domestic birds [1]. The recurrent emergence of HPAI highlights the ongoing challenge associated with its control at an international scale. Of particular concern is its recent expansion in host range, with infections reported in several mammalian species, including dogs and dairy cattle, indicating a potential shift in its epidemiology and ecology [2].

Although Morocco remains free from HPAI, it faces a substantial risk of introduction due to its geographical position along major migratory wild bird flyways linking Europe and Africa, as well as its extensive commercial exchanges. These factors may facilitate the introduction and dissemination of the virus through both wild and domestic pathways [3]. The potential impact of an outbreak would be considerable, given the economic importance of the poultry sector, which generates approximately 36.9 billion Dirhams in revenue and accounts approximately 23.3 million working days annually [4]. In regions where HPAI is endemic or epidemic, the virus is frequently spread through the movement of live birds, poultry products, and related equipment [5,6,7]. In this context, a thorough understanding of disease transmission dynamics is essential for strengthening surveillance and improving control strategies. The analysis of mobility networks within the poultry sector provides a powerful framework for investigating the mechanisms that drive the spread of infectious diseases. To better access the potential dissemination of HPAI in Morocco, it is crucial to characterize the structure and intensity of movement networks linking poultry farms and associated service facilities.

Accordingly, the present study applies social network analysis to collect and analyze data on transport vehicle movements across key components of the poultry sector in the Casablanca-Settat region. This region constitutes a particularly relevant study area, as it accounts for approximately 45% of the national annual white meat production [8]. Morocco’s poultry sector has experienced substantial growth over recent decades, with production units now established across nearly all provinces of the country. The sector currently comprises more than 10,000 farms, 40 hatcheries, and 28 feed mills [4].

We hypothesized that poultry transport movements in the Casablanca-Settat region are structured around a small number of highly connected municipalities (nodes) and structurally critical connectors, whose removal would disproportionately fragment the network compared with random node removal, and that these nodes should therefore be prioritized for HPAI surveillance.

Within this framework, the objective of this study is to characterize the structure of the live poultry transport network in the Casablanca-Settat region and to identify the municipalities and connections that occupy structurally critical positions within it. As the study is cross-sectional and based on declared movement data, and as no outbreak records or epidemiological simulations were analyzed, the findings describe potential pathways for pathogen dissemination and the relative structural exposure of network components.

## 2. Materials and Methods

### 2.1. Study Area

The study was conducted in the Casablanca-Settat region, located in northwestern Morocco.

This region holds a strategic position within the Moroccan poultry sector, producing 310,000 tons of poultry meat annually, which represents 45% of national production, and supplying 30% of the country’s table eggs [8]. The region also hosts Morocco’s two largest wholesale markets for live poultry and table eggs, consolidating its role as the primary hub for poultry trade and distribution at the national level. Consistent with findings from comparable settings, live animal markets have been identified as high-risk nodes for the transmission and amplification of infectious pathogens, particularly highly pathogenic avian influenza (HPAI) [5,9].

The Casablanca-Settat region is one of the areas in Morocco at the highest risk of the emergence and spread of highly pathogenic avian influenza (HPAI). A recent study integrating geographic information systems (GIS) and multi-criteria decision analysis (MCDA) enabled the development of a spatial risk model for HPAI dissemination across the country. This model identified the provinces of the Casablanca-Settat region and its adjacent territories as having the highest risk levels in the country [10].

This heightened vulnerability profile is due to the presence of several risk factors: a high concentration of poultry farms, intense live poultry trade and commercial activity, high-throughput wholesale markets that could facilitate the spread of the virus, and proximity to migratory bird flyways, which could introduce and spread the virus regionally and internationally [1,11,12].

All of these factors make a strong case for choosing this region as the main study area for investigating the role of poultry transport networks (including the movement of live birds, poultry feed, table eggs, and hatching eggs) in determining HPAI transmission dynamics. Numerous studies have demonstrated that the movement of poultry and poultry products between the various structures of the supply chain constitutes a major driver of avian influenza dissemination, encompassing both highly and low pathogenic strains [5,13,14].

In this context, the survey conducted by Boudouma et al. (2025) [10] supported this finding by assigning poultry transport the highest risk score among all evaluated factors, thereby underscoring its predominant role in the potential spread of the virus within the region.

### 2.2. Sampling Methods

This study considered only farms authorized by the competent sanitary authority, the National Office for Food Safety (ONSSA). Within the administrative region of Casablanca-Settat, a total of 2445 authorized farms were identified, distributed across 138 municipalities spanning 9 provinces.

To ensure sufficient geographical coverage and statistical representativeness of the farm population, the study adopted a stratified random sampling approach. This technique involves partitioning the target population into distinct, internally homogeneous subgroups, known as strata, based on common characteristics. This yields to more precise and representative estimates than what simple random sampling alone would provide [15].

The present study used two complementary variants of this method. The first was a disproportionate approach, designed to ensure the representation of municipalities across different levels of farm concentration with greater weight assigned to areas of high-density poultry farming. The second was a proportional approach, aimed at reflecting the distribution of the different production types present within the study region.

**Figure 1:**
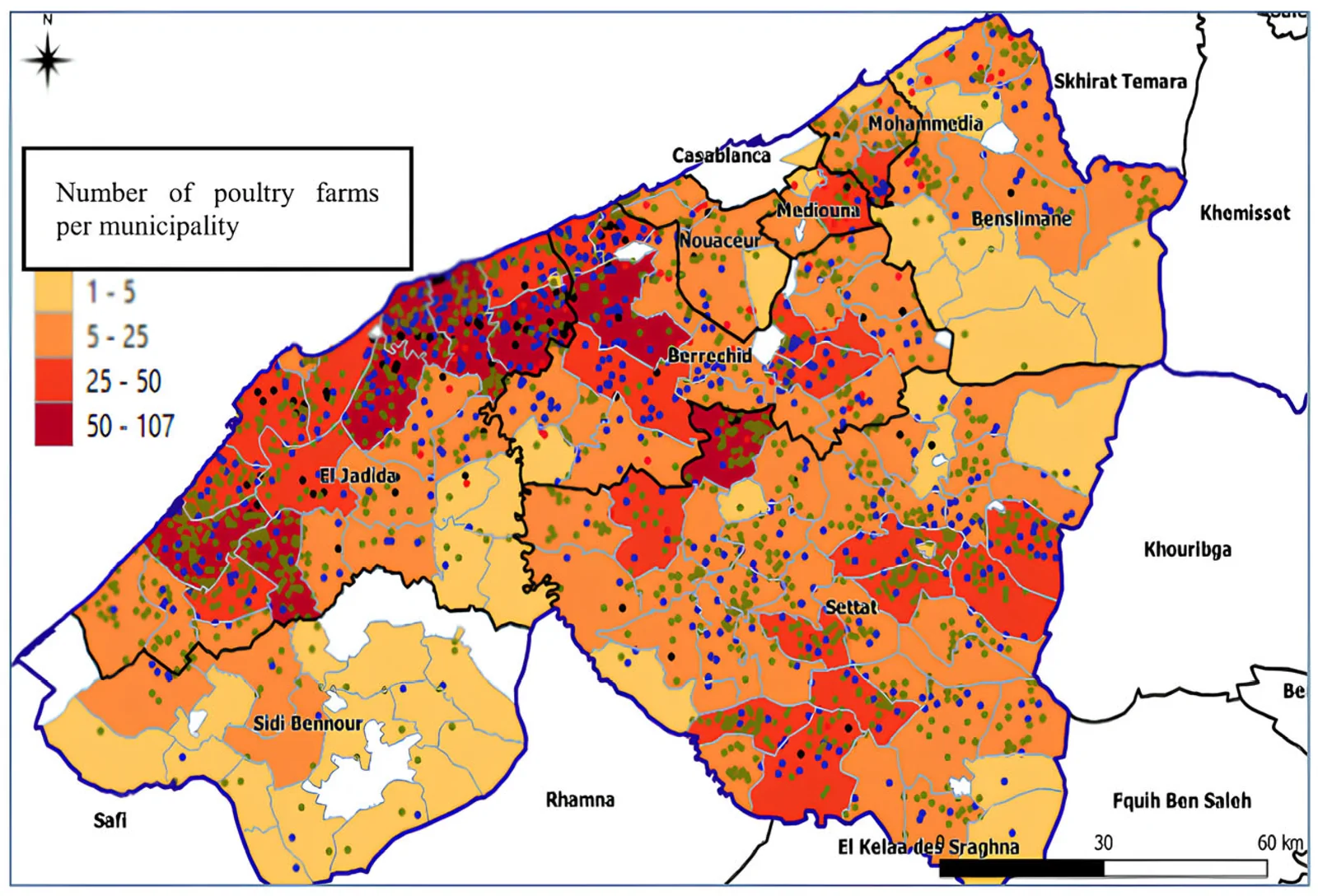
Spatial distribution of poultry farms across municipalities in the Casablanca-Settat region, Morocco. Poultry farms are represented according to production type: broiler farms (green dots), meat turkey farms (blue dots), laying hen farms (red dots), and breeder chicken farms (black dots).

Based on the intensity of poultry farming activity, municipalities within the study region were classified into four distinct strata (Figure 1).

Unlike livestock sectors subject to pronounced seasonal cycles, poultry production and marketing activity in Morocco occurs at a relatively constant pace throughout the year; the two field survey periods were therefore treated as consecutive components of a single continuous data-collection effort, and movements recorded across both were pooled into a single network for analysis.

Sampling was intentionally disproportionate to align with the primary objective of the survey, which focused on poultry mobility patterns. Areas with the highest farm concentrations were expected to generate the largest volume of movements; therefore, municipalities in the “Very high” density stratum were oversampled, with the number of sampled municipalities doubled relative to other strata. For the remaining three strata, a uniform sampling rate of 25% of municipalities was applied (Table 1).

**Figure 2:**
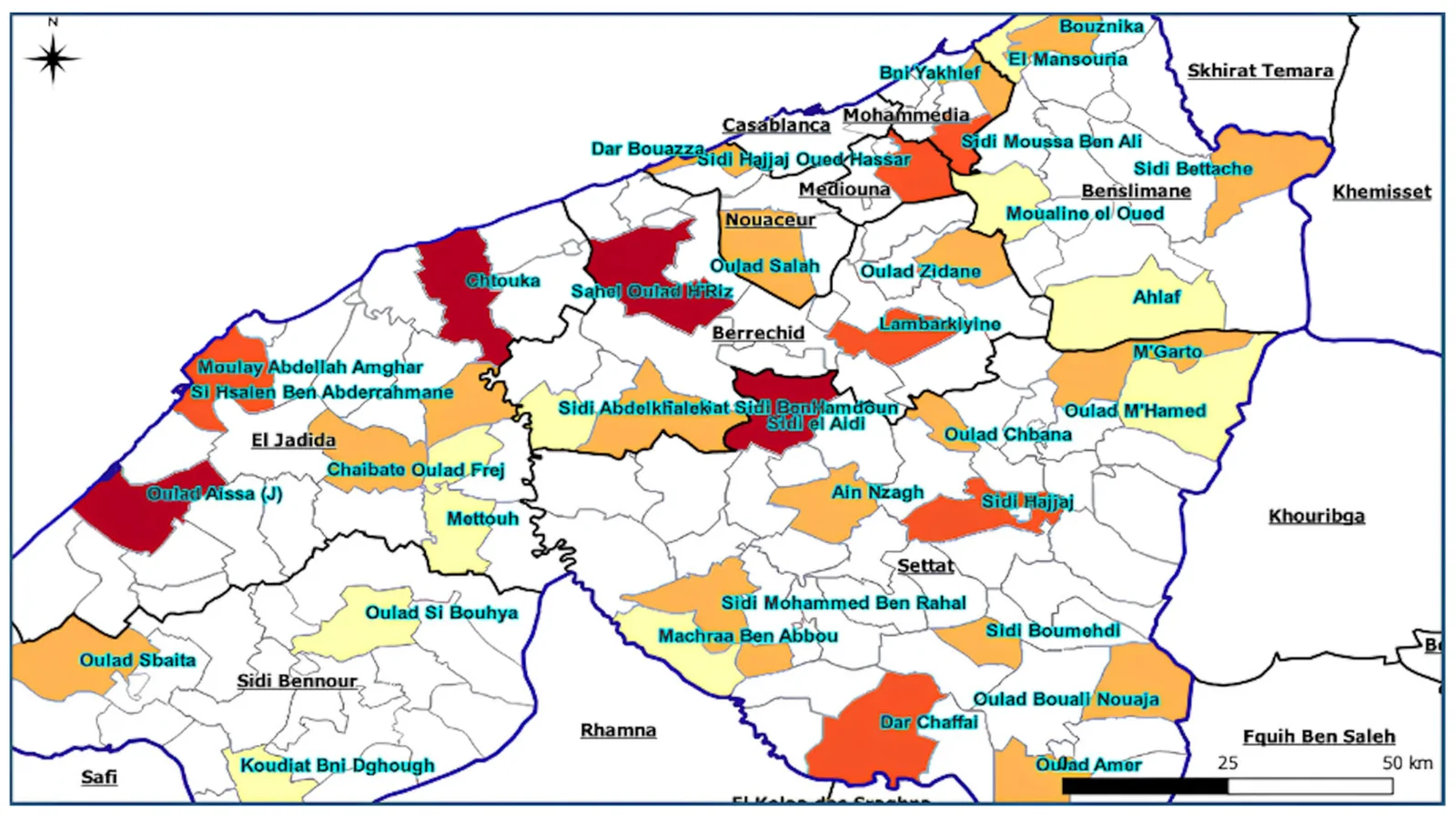
Sampled municipalities within the study area.

The sampled municipalities in the four strata are shown in Figure 2.

The overall sampling coverage of municipalities within the study area was 26.8% (37/138). The minimum required sample size was estimated at 333 farms and subsequently increased to 370 farms to account for potential non-responses. The final sample was allocated proportionally across the 37 sampled municipalities, based on the number of poultry farms present in each municipality.

Given the complexity of the poultry production chain and the diversity of its distribution channels, data collection was conducted during two distinct field survey periods. These surveys involved a broad range of stakeholders including poultry farmers, private veterinarians, traders, and other key actors within the sector, to obtain comprehensive information on mobility networks and movement patterns throughout the poultry industry. This approach enabled a detailed characterization of interactions and movements that may contribute to the dissemination of infectious diseases.

### 2.3. Field Investigations and Data Collection

An Origin–Destination questionnaire was developed to collect data on mobility networks in the poultry sector within the study area. It covered the municipalities of origin and destination of movement and the nature of places involved, such as wholesale markets, farms, hatcheries, feed mills, weekly souks, and slaughterhouses. It also addressed the nature of the transported products such as broilers, laying hens, table eggs, breeders, turkeys and hatching eggs. The questionnaire was anonymous and short, limited to one line for each respondent. The questionnaire was first tested on private veterinarians and members of the Interprofessional Federation of the Poultry Sector (FISA) to validate its use in the field. This pre-test aimed to evaluate the clarity of the questions, the relevance and confidentiality of the information requested, the time required to complete the questionnaire and the degree of involvement of the targeted respondents.

Based on the list of sampled municipalities, field visits were conducted to collect the necessary data using the previously established and validated questionnaire. The latter was given to the private veterinarians in charge of the poultry farms as well as directly to farmers in the sampled municipalities. The primary objective was to collect inflows and outflows at the farm level by tracking the origins and destinations of movements involving day-old chicks, poultry feed, table eggs, and poultry destined for slaughter. Complementary surveys were also carried out in the wholesale markets for live poultry and table eggs in Casablanca, with a view to capture flow dynamics at this level of the distribution chain. The data-collection process lasted for six months and consisted of face-to-face interviews with private veterinarians, farmers, and traders in the wholesale markets, with the aim of tracing the origins and destinations of transport routes registered at the municipality level.

This integrated approach involving private veterinarians and professionals of the poultry sector has enabled the collection of data on movement patterns at farm level. The surveys in the wholesale markets of Casablanca, on the other hand, provided additional perceptions into the mobility networks specific to the sector, allowing for an overview of the marketing and distribution circuits. However, it is important to take into account that the complex structure of these circuits, together with their often-irregular nature, constituted a methodological limitation in the interpretation of the collected data.

### 2.4. Analytical Method

Social network analysis (SNA) is a powerful method for identifying and quantifying links between actors within a movement network. Its earliest use in human epidemiology was in the study of sexually transmitted diseases, when Klovdahl (1985) showed that several hundred individuals were connected, directly or indirectly, through a network model [16]. The paradigm established by that work has since been extended to a wide range of pathogens and contact types [17]. Since the 2000s, especially following the foot-and-mouth disease epizootic in Great Britain, network analysis has been more and more used in veterinary epidemiology, mainly for studying livestock movement and to predict the potential spread of infectious diseases [18,19,20]. The U.S. National Research Council, network science defines this method as “the study of network representations of physical, biological, and social phenomena leading to predictive models of these phenomena” [21]. Social Network Analysis (SNA) is a useful methodological tool in animal epidemiology, providing a deeper understanding of the consequences of animal movements and the resulting contact networks on the dynamics of animal disease spread. Moreover, its inherent flexibility allows for the integration of diverse types of contacts, thereby enriching and increasing the analytical scope of this method. With the availability of more and more data, SNA is poised to become an even more powerful tool for the anticipation and management of animal disease outbreaks, providing critical support to epidemiologists and decision makers in their efforts to prevent and control infectious diseases.

The social network analysis framework applied in this study followed the methodological principles described by Dubé et al. (2009) [18], using centrality measures, including in-degree, out-degree, and betweenness, to identify key nodes within the poultry movement network of the Casablanca-Settat region.

Social network analysis enables the computation of a large set of indicators that describe the structure of the network and rank the nodes according to their relative influence [22]. While various measures exist, only those employed in the present study are described here. We can divide these measures into global and local measures, capturing both overarching network structure and node-specific properties [23].

#### 2.4.1. Global Measures

Global measures describe the network in its entirety by quantifying statistical properties emerging from its overall architecture. Rather than looking at individual nodes in isolation, they provide a collective reading of all nodes and edges, revealing patterns of connectivity and cohesion at the network level. Global measures are particularly valuable for understanding how the structural organization of a network may influence the dynamics of disease transmission [24,25,26].

#### 2.4.2. Local Measures

Local measures focus on the properties of individual nodes and their immediate connections. These measures capture structural characteristics at a finer scale, providing detailed information about the immediate environment of each node [19,27]. The primary local measures used in this study include:

- Centrality measures: Quantify the influence or importance of a node within the network. In epidemiology, centrality metrics are widely used to identify key individuals, nodes, or actors that may play critical roles in disease transmission.
- Cohesion measures: Assess the degree to which nodes form tightly connected subgroups, revealing clusters of individuals or actors with strong and cohesive relationships.
- Centralization indices: To evaluate the significance of the observed network structures, each network’s parameters were compared with the mean values derived from 100 random generated networks of the equivalent size using the Erdős–Rényi model. These random networks served as a baseline, allowing assessment of whether the observed structural patterns were statistically meaningful or could arise by chance. Substantial deviations of centralization values from those of random networks indicate the presence of distinct structural features within the network, which may influence the dynamics of HPAI transmission [28]. In this study, the centralization indices analyzed included in-degree centralization, out-degree centralization, and betweenness centralization.

### 2.5. Data Management and Network Analysis

Collected mobility data were entered into a Microsoft Excel database, with an origin municipality and a destination municipality and then converted into movement using a single line after removing duplicates.

The 37 municipalities constituting the sampling units are the locations at which respondents were interviewed. The nodes of the resulting network are the municipalities of origin and destination declared by these respondents, which explains why the network comprises 126 nodes: municipalities that were not themselves sampled enter the network whenever they were reported as an origin or a destination of a declared movement.

Edges were treated as binary rather than frequency-weighted because precise movement counts between municipality pairs could not be reliably ascertained through the questionnaire survey, and because the analytical objective was to characterize network connectivity structure—which locations are linked, and how these links shape hub position and vulnerability to fragmentation—rather than to quantify contact intensity. The networks were therefore analyzed as unweighted networks, with each edge contributing equally to the network topology irrespective of the number of poultry movement involved.

To assess the robustness of the centrality-based ranking to sampling variation, a leave-one-node-out (jackknife) stability analysis was performed. Each of the 126 municipalities was successively removed from the network, and betweenness centrality was recalculated for the resulting 125-node network. The resulting top-15 ranking and the identity of the top-3 hub municipalities were compared with those obtained from the full network, and the proportion of iterations in which they remained unchanged was recorded.

Cartographic analysis was conducted using Quantum GIS (QGIS) software, version 3.22. Each municipality was mapped using coordinates centered on the centroid of its polygon under the EPSG:4326 (WGS 84) reference system.

Microsoft Excel was used for preliminary statistical analyses, while R (ersion 4.4.1) and RStudio (version 2024.04), with the “sna” (version 2.7) and “igraph” (version 2.0.1) packages, were used for network analysis and the calculation of various measures. The analytical framework proceeded through the following steps:

1. Descriptive Characterization of the Network

First, global descriptive parameters of the network were computed. Density reflects the proportion of observed ties relative to the total number of ties theoretically possible within the network. Diameter corresponds to the maximum geodesic distance between any two nodes, while average path length represents the arithmetic mean of all pairwise inter-node distances. The Kappa heterogeneity index quantifies the variability in node connectivity across the network, and centralization indices assess the degree to which connections are concentrated around specific nodes.

2. Computation of Centrality Indices

Second, centrality indices were calculated at the individual node level. In-degree refers to the number of incoming ties received by a given node, whereas out-degree reflects the number of outgoing ties it generates. Betweenness centrality measures the frequency with which a node serves as an intermediary along the shortest path connecting two other nodes within the network.

3. Cohesion Analysis and Community Detection

Third, cohesion indicators were computed to identify cohesive subgroups within each network—that is, clusters of nodes maintaining particularly dense and structured relationships among themselves. In parallel, community detection was performed. Communities are defined as groups of nodes more densely interconnected with one another than with the remainder of the network, thereby revealing substructures likely to shed light on epidemic propagation dynamics.

Definitions and interpretations of all parameters are provided in the Appendix A. This methodological framework enables a rigorous characterization of the structure and dynamics of mobility networks, by identifying vulnerability hotspots and cohesive subgroups, thus providing critical insights for the management and prevention of HPAI outbreaks in the Casablanca-Settat region.

## 3. Results

### 3.1. Statistical and Cartographic Analysis of Transport Movement

**Figure 3:**
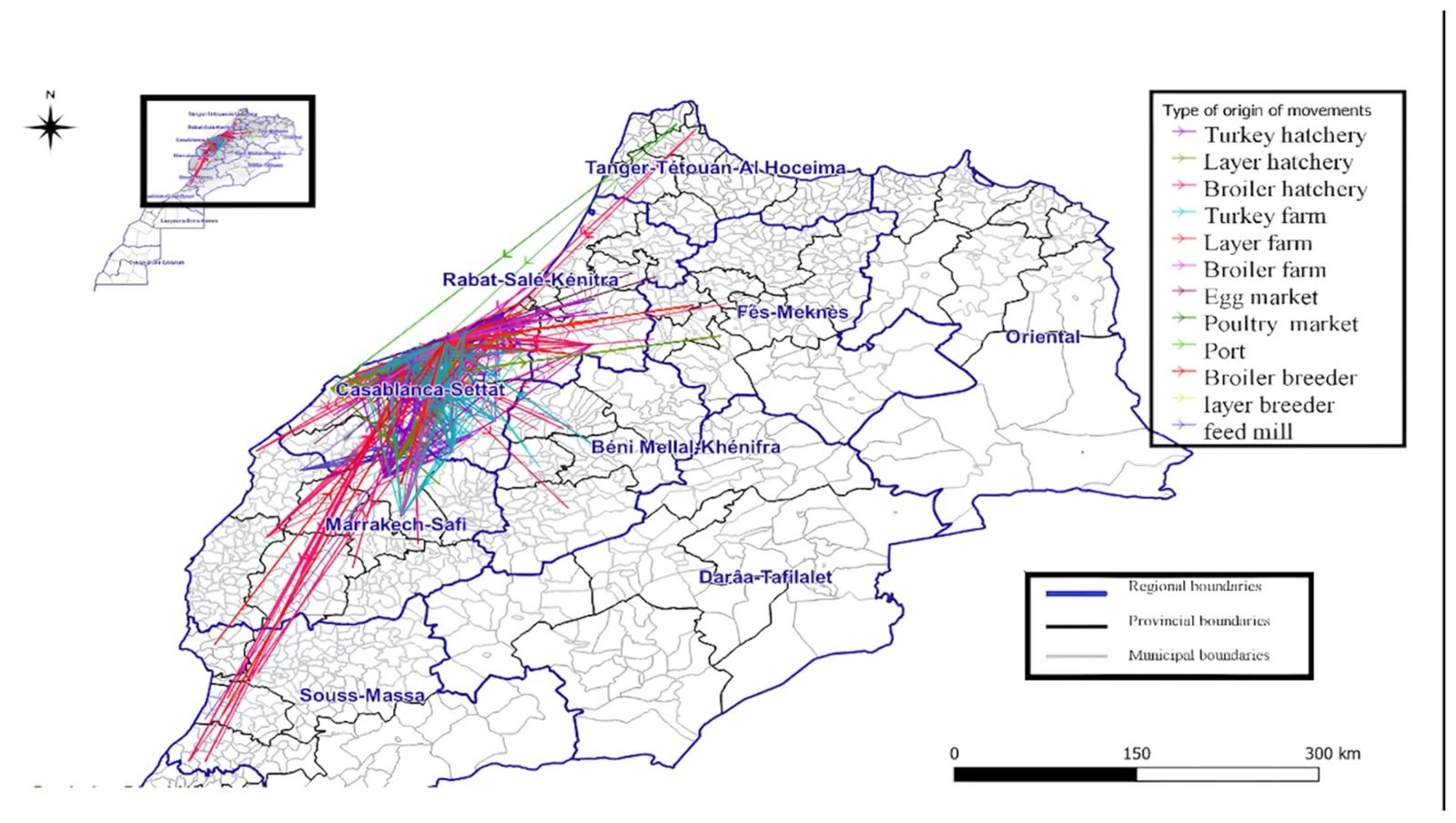
Map of the transport network within the study region and its connections with other regions.

According to survey responses, a total of 945 transport movements involving 126 municipalities were recorded. Among these, 65 municipalities were located within the study region, and 61 municipalities were located outside of the region, distributed over 18 provinces and five administrative regions of the country: Fès-Meknès, Marrakech-Safi, Souss-Massa, Béni Mellal-Khénifra, and Tanger-Tétouan-Al Hoceima. These movements are indicative of the broader spatial extent of poultry transport flows connecting the study region of Casablanca-Settat with other regions of Morocco (Figure 3).

The transport movement concerned various products, with live poultry accounting for more than 44% of all movements, followed by day-old chicks at 30% of routes. Poultry feed and table eggs represented smaller proportions, accounting for 15% and 10% of routes, respectively, whereas hatching eggs constituted a marginal share of 1%.

Hay Mohammadi in Casablanca and Had Soualem played a central role, accounting for 14.6% and 14.5% of total movements, respectively. Other key municipalities included Sahel OuladH’Riz (6.03%) and Labrikiyine (2.33%), demonstrating their importance in the transport network.

### 3.2. Global Transport Network Analysis

The overall transport network comprised 126 nodes (municipalities) connected by 376 links representing transport movement, of which only 25.9% were bidirectional (reciprocity), meaning that potential contamination could occur in both directions for only 25.9% of routes (Table 2).

The network exhibited a low density (0.023), indicating that only a small proportion of all possible connections between nodes were present. However, the kappa heterogeneity coefficients associated with node activity were all substantially greater than 1 (κDegree = 4.47, κIndegree = 4.86, and κOutdegree = 5.74), revealing a highly heterogeneous network structure. This suggests that while most nodes maintained relatively few connections, a limited number of nodes acted as highly connected hubs.

Furthermore, the network diameter was six, indicating that the longest shortest path between any two connected nodes consisted of six steps. This relatively small diameter highlights the potential for rapid dissemination of pathogens across the network despite its low overall connectivity.

The network exhibited a low density of 0.023. However, the kappa heterogeneity coefficients related to node activity parameters were all well above 1 (Kappa Degree = 4.47; Kappa In-degree = 4.86; Kappa Out-degree = 5.74), indicating that the network was heterogeneous. The network diameter was equal to six.

The centralization indices of the observed network were compared with those of 100 Erdős–Rényi random networks generated with the same number of nodes and edges. The observed in-degree, out-degree and betweenness centralization indices were 0.358, 0.437 and 0.0029 respectively, against random-network means (±SD) of 0.039 ± 0.007, 0.042 ± 0.008 and 0.0006 ± 0.0001 (Table 3). None of the 100 simulated networks attained the observed centralization on any of the three measures (*p* < 0.001 in each case). The hub-dominated structure observed therefore reflects genuine degree heterogeneity in the transport network rather than an artifact of network size or of the sampling design.

### 3.3. Centrality Parameters

#### 3.3.1. Total Degree

The global activity indicator, degree, showed that the most active nodes in the region were Had Soualem (degree = 95), Hay Mohammadi (degree = 63), and Sahel OuladH’Riz (degree = 40), followed at a distance by Labrikiyine (degree = 21), Settat (degree = 21), and Cherrat (degree = 20) (Table 4).

Across all 126 leave-one-out iterations, the top-15 ranking showed a mean overlap of 98.5% with the original ranking (SD = 4.2%; range 73.3–100%), and the composition of the top-15 changed at all in only 20 of the 126 iterations (15.9%). The three highest-ranking hub municipalities (Had Soualem, Hay Mohammadi and Sahel Oulad H’Riz) remained identical after removal of any other single municipality in 96.0% of iterations (mean overlap 2.96/3), and each was retained within the top-15 in 100% of iterations.

#### 3.3.2. In-Degree and Out-Degree

The in-degree parameter revealed marked differences in the level of exposure among nodes. In particular, Hay Mohammadi (in-degree = 46) and Had Soualem (in-degree = 37) appeared to be the most vulnerable to pathogen introduction due to the large number of distinct origins with which they were connected. These were followed by Sahel Oulad H’Riz (in-degree = 20), Settat (in-degree = 19), and Sidi Bou-Othmane (in-degree = 12). Nodes characterized by a relatively high in-degree and a low out-degree may be particularly susceptible to local pathogen persistence. For example, Sidi Bou-Othmane (out-degree = 1) receives connections from multiple sources while maintaining very limited outward movements, potentially favoring the local retention and circulation of infection.

**Figure 4:**
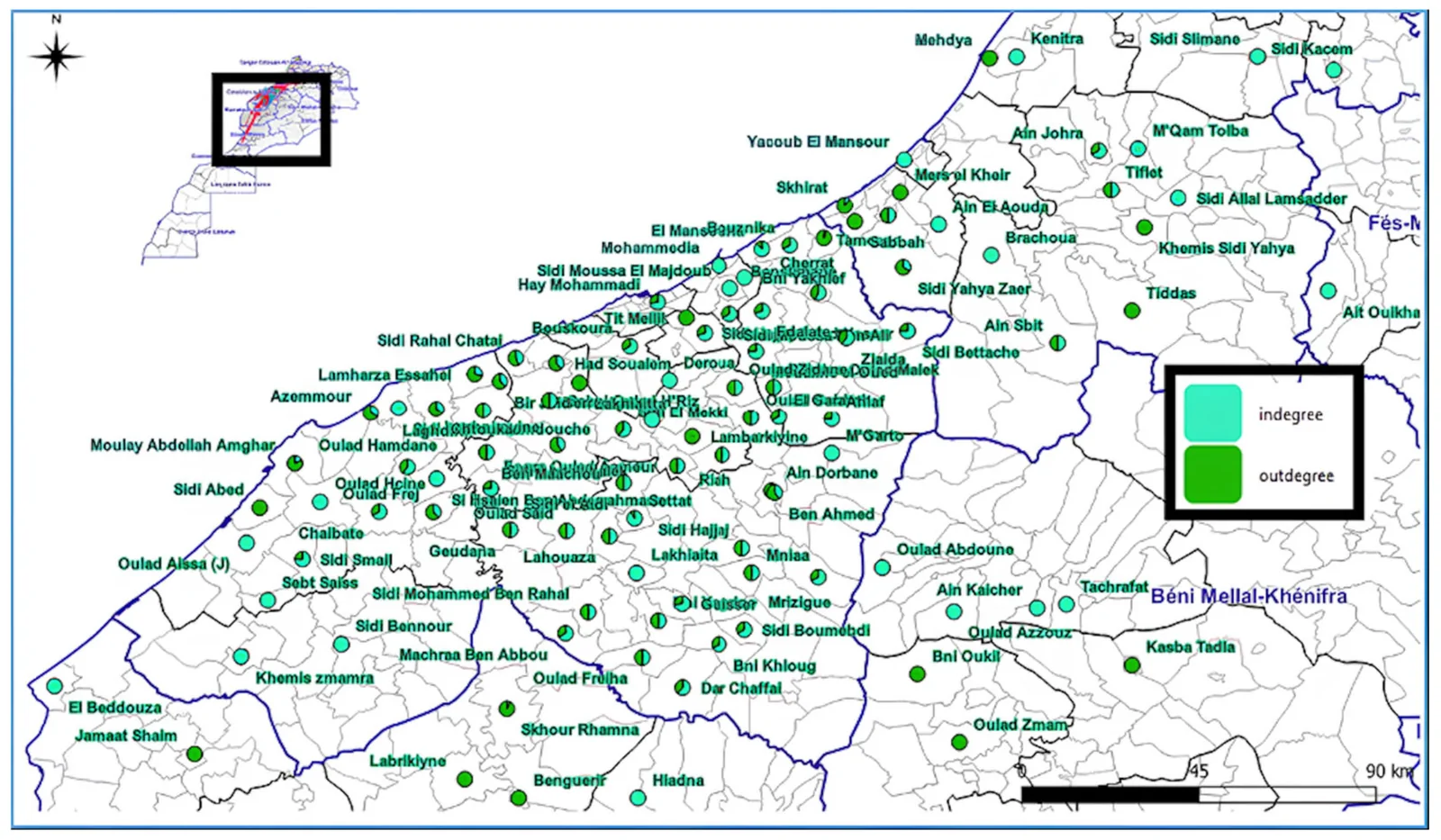
Representation of the in-degree and out-degree centrality parameters (zoom on the study region).

The out-degree parameter highlighted nodes with a high potential to disseminate infection throughout the network. Had Soualem (out-degree = 58), Labrikiyine (out-degree = 21), Sahel Oulad H’Riz (out-degree = 20), Cherrat (out-degree = 19), and Hay Mohammadi (out-degree = 17) exhibited the highest numbers of outgoing links. Owing to their extensive connectivity with multiple destinations, these nodes could facilitate the rapid dissemination of pathogens during an outbreak. Notably, some nodes combined both high susceptibility and high transmission potential. Sahel Oulad H’Riz, for example, displayed equally high in-degree and out-degree values (20 and 20, respectively), indicating a dual role as both a major recipient and distributor of movements within the network, thereby representing a critical node for disease surveillance and control interventions (Figure 4).

#### 3.3.3. Betweenness

**Figure 5:**
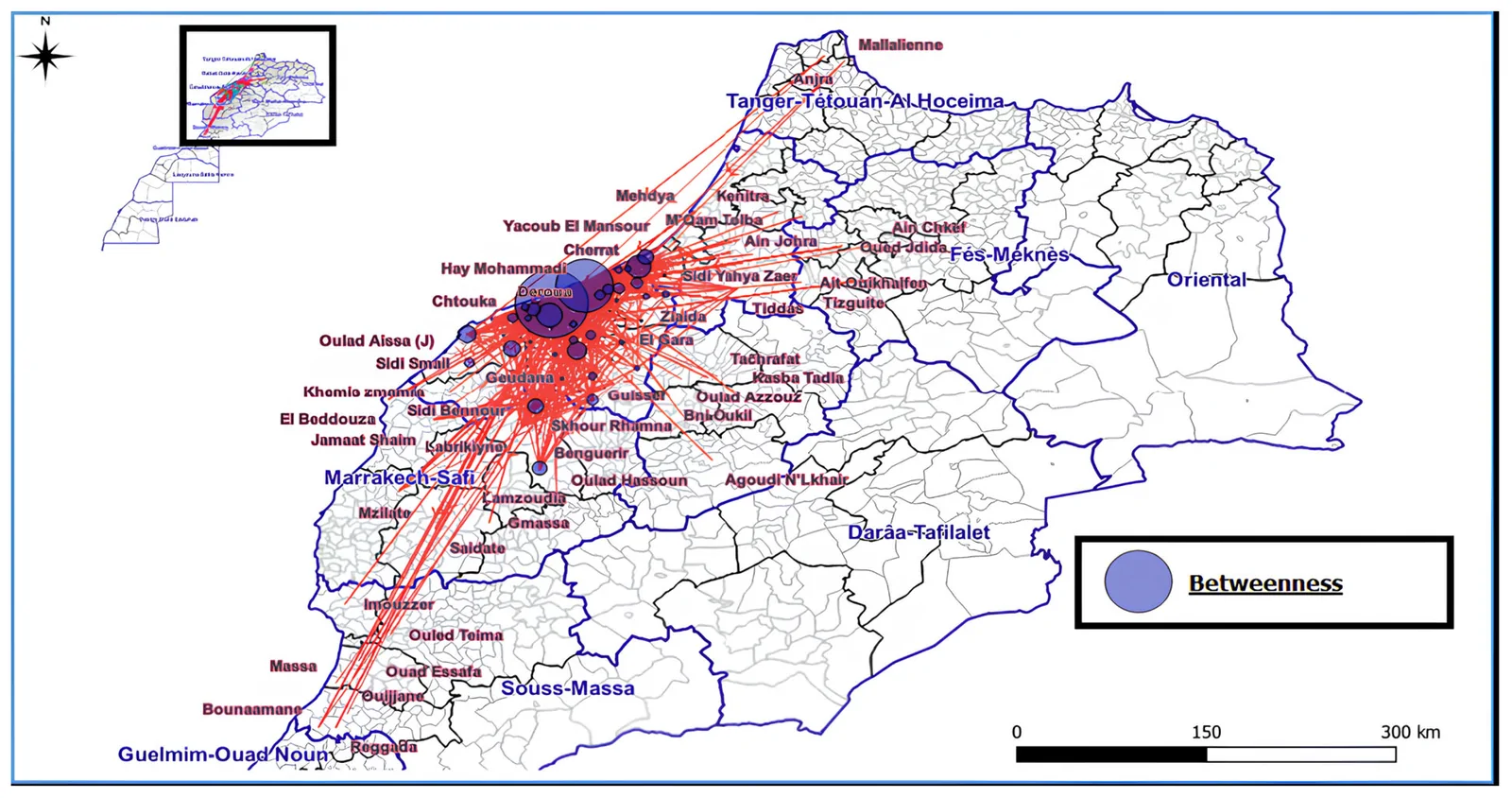
Overall mobility network of transport means within the poultry sector showing the betweenness parameter.

Had Soualem (betweenness = 5863.62) was by far the main indispensable hub in the network within the study area. It played the role of a bridge facilitating the passage of flows among many municipalities that would otherwise not be connected without passing through it. It was followed, at a considerable distance, by Hay Mohammadi (betweenness = 3601.05), which represented only about two-thirds (61.4%) of Had Soualem’s value. Sahel OuladH’Riz (betweenness = 725.67) and Cherrat (betweenness = 653.63) represented only 12% and 11%, respectively, of Had Soualem’s betweenness value, but nevertheless remained among the most influential municipalities in terms of connecting the different subnetworks of the graph compared with the other municipalities in the study area (Figure 5).

### 3.4. Strong Component

**Figure 6:**
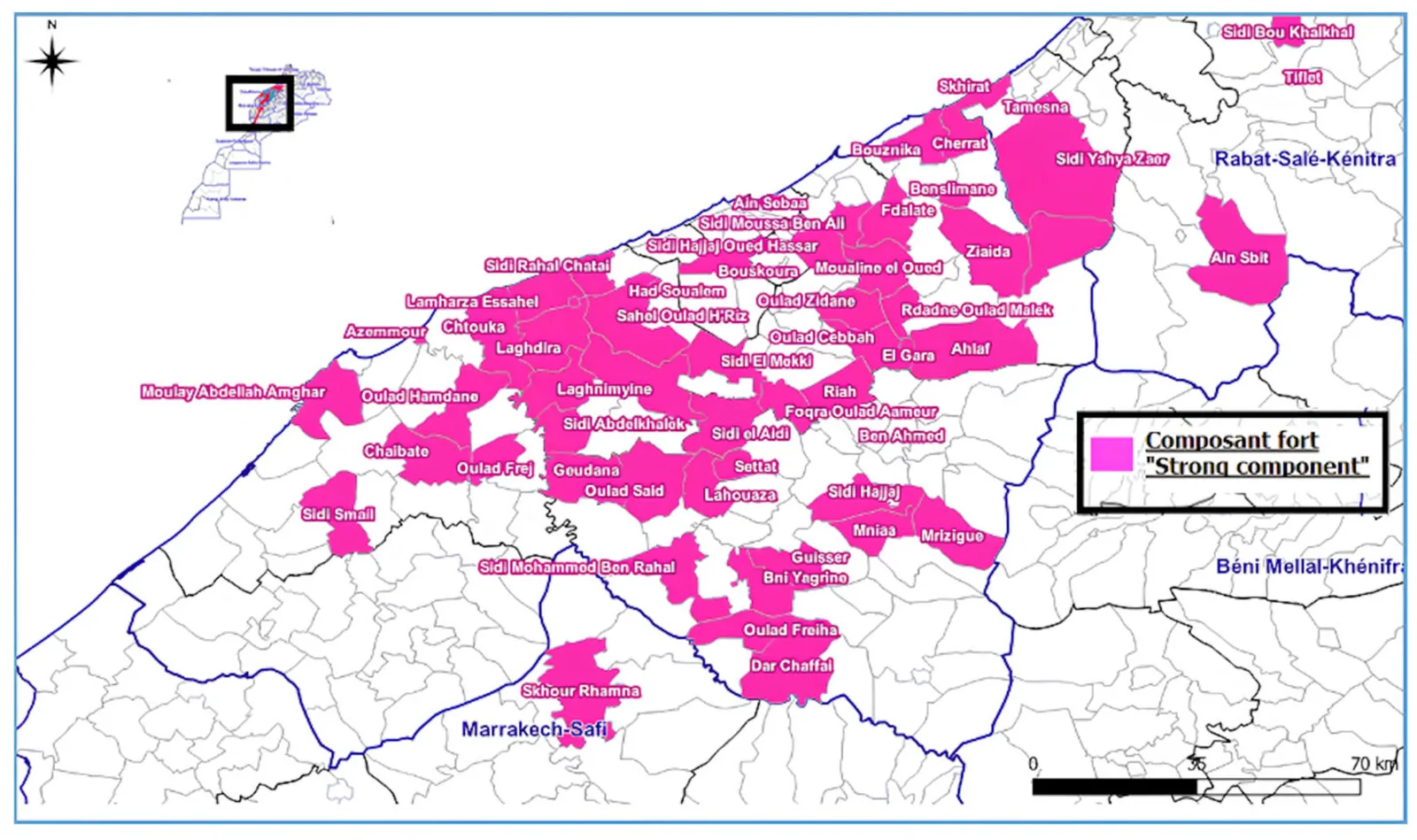
Extent of the strong component of the poultry network in the Casablanca-Settat region and its connections.

The network contained one strong component comprising 57 municipalities strongly connected through movements, such that all of them could be reached from one another. This strong component extended across 11 provinces (Berrechid, Casablanca, Settat, Rhamna, Benslimane, El Jadida, Mohammedia, Mediouna, Khémisset, Nouaceur, and Skhirat-Témara) in three different regions of the Kingdom: Casablanca-Settat, Rabat-Salé-Kénitra, and Marrakech-Safi (Figure 6).

### 3.5. Articulation Points (Cutpoints)

**Figure 7:**
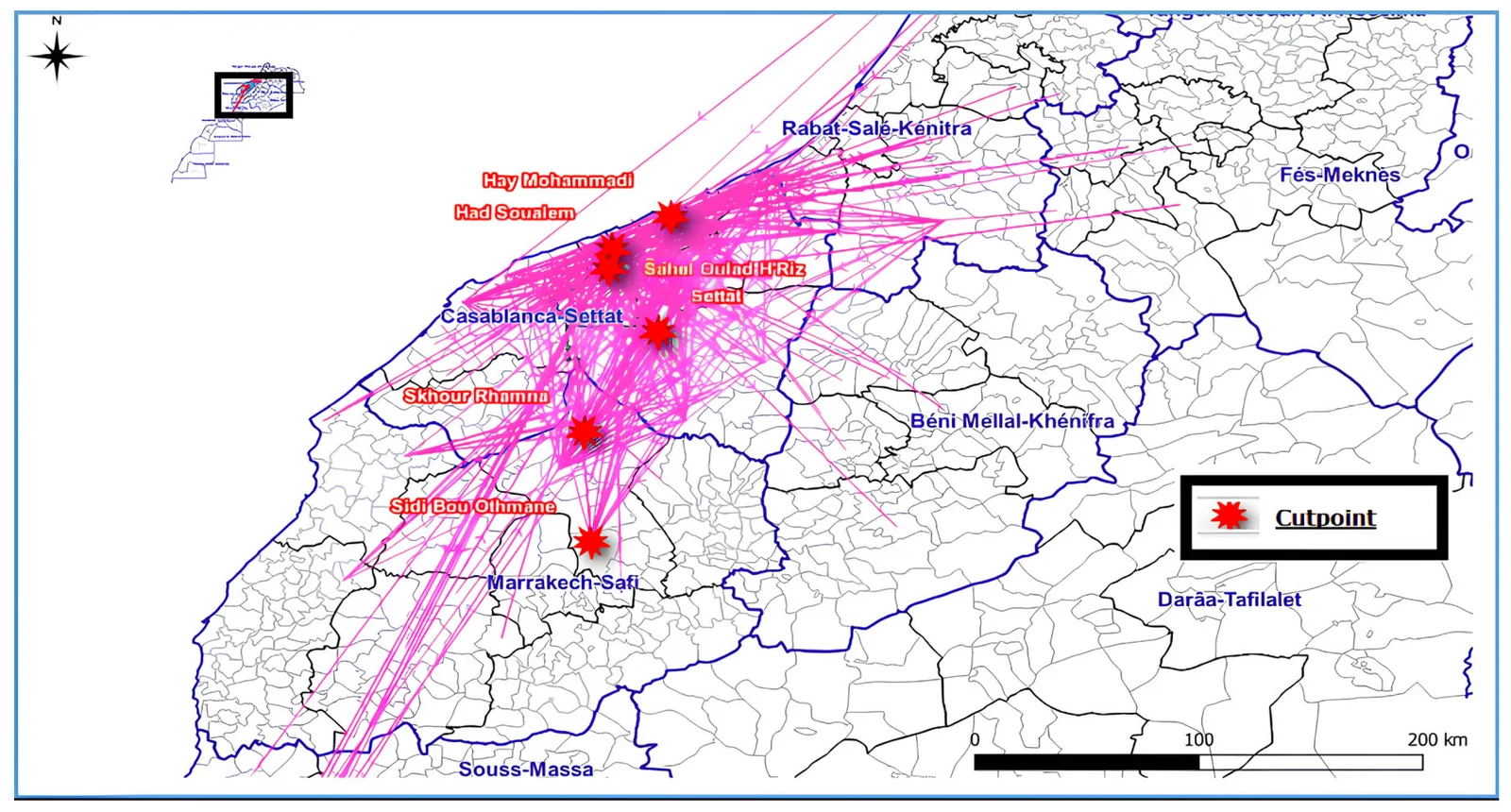
Articulation points (“cutpoints”) in the poultry mobility network of the Casablanca-Settat region and its connections.

Cutpoints are nodes whose removal increases the number of network components and thus disrupts disease spread among the various parts of the network. Six municipalities were identified as articulation points, or cutpoints: Hay Mohammadi, Had Soualem, Sahel OuladH’Riz, Settat, SkhourRhamna, and Sidi Bou Othmane (Figure 7).

Preventive and control measures applied at these nodes could help control, or even stop, the spread of the epidemic to other parts of the network.

### 3.6. Network Resilience

**Figure 8:**
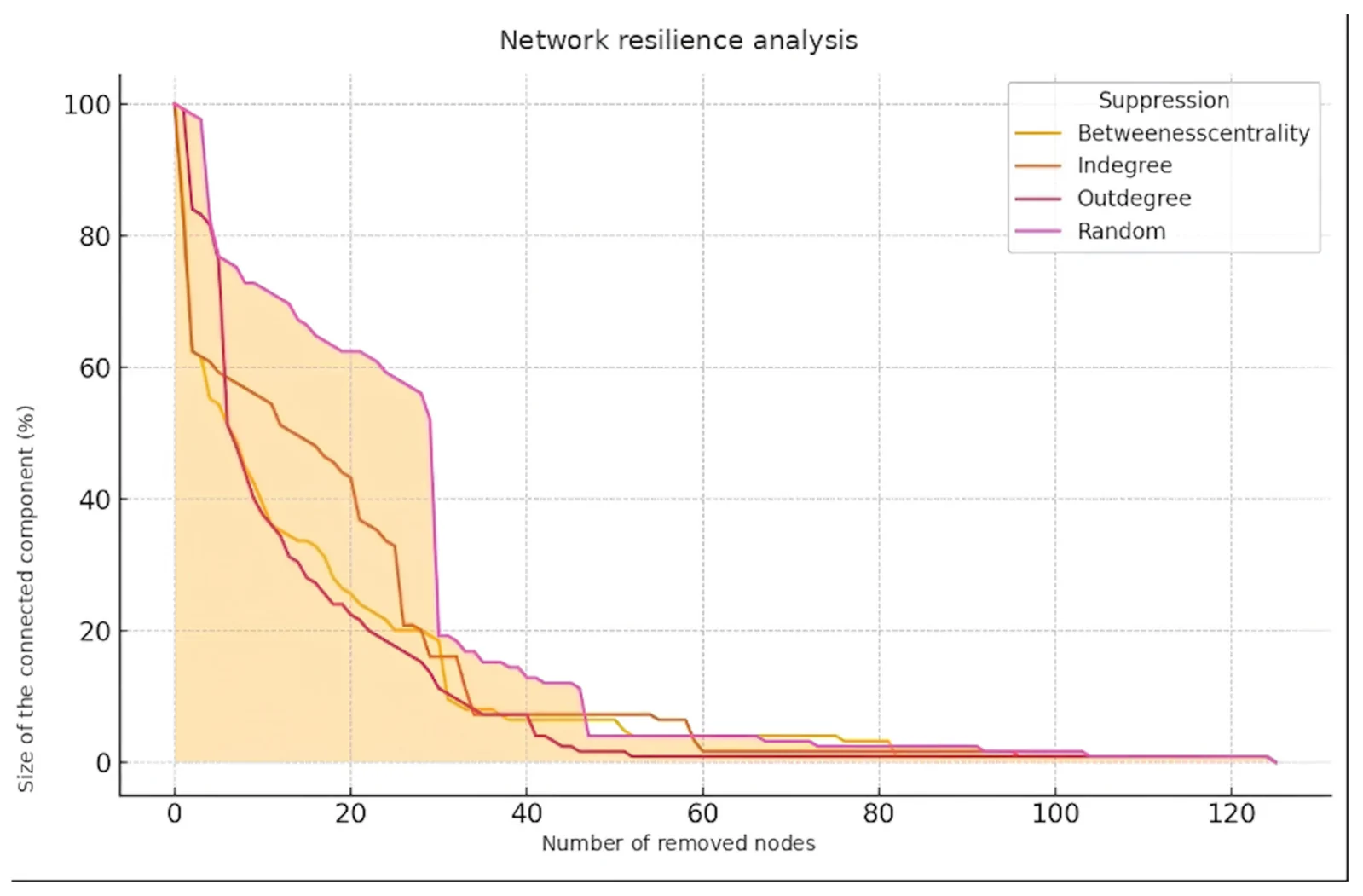
Network resilience under targeted and random node removal. The targeted-removal curve is deterministic (nodes removed in decreasing order of betweenness centrality). The random-removal curve represents the mean of *N* independent simulations; the shaded band indicates the 2.5–97.5 percentile envelope across simulations.

We measured network resilience by measuring the impact of targeted node removal on network connectivity. Resilience is the capacity of a network to preserve its structure and functionality under targeted attack. Figure 8 shows the robustness of the global mobility network to different node removal strategies. The y-axis is the size of the largest connected component (in percentage), and the x-axis the number of nodes removed.

## 4. Discussion

The mobility network analysis of the poultry sector in the Casablanca-Settat region shows a heterogeneous structure (heterogeneity coefficient kappa greater than 1), in which a small number of nodes monopolize the majority of contacts while most other nodes maintain only a limited number of connections. The significant level of clustering combined with long-distance contacts makes this network easily navigable for pathogens. Heterogeneous networks are known to facilitate early disease emergence and highly virulent spread, but usually over a shorter period than other types of networks [26].

The degree distribution of the network is highly heterogeneous: a small number of municipalities concentrate a disproportionate share of connections, while the majority maintain few. This heterogeneity, quantified by an in-degree centralization of 0.358 and an out-degree centralization of 0.437 against random-network values of 0.039 ± 0.007 and 0.042 ± 0.008 respectively, is consistent with observations reported for other livestock movement networks [29]. Napp et al. (2018) identified it as a determinant of the vulnerability of European poultry systems to the rapid spread of HPAI H5N8 during the 2016–2017 epizootic [7], and Hinjoy et al. (2024) reported a comparable concentration of trade connectivity in smallholder poultry trade networks in the border provinces of Thailand [30], indicating that this structural pattern is not specific to a single production context or period. Moreover, Kurscheid et al. (2017) [31], through their investigation of live bird market networks in Bali and Lombok, Indonesia, demonstrated that poultry trade networks exhibit strong connectivity heterogeneity, whereby the risk of HPAI H5N1 transmission is concentrated on a small number of nodes with high degree and high betweenness centrality. A critical finding of these authors was that such networks display considerable resilience to random control measures, given that the vast majority of nodes maintain only a few links, rendering their random removal largely ineffective in disrupting overall network integrity. Conversely, these same networks prove highly vulnerable to targeted control strategies directed at hub nodes, as the disruption of a limited number of highly connected nodes can substantially fragment the network and impede pathogen spread [31].

These findings carry direct relevance to the Casablanca-Settat context, where a comparably structured network underscores the need for prioritized, risk-based surveillance and intervention strategies focused on the most epidemiologically influential nodes.

The network diameter of 6 indicates that the most distant nodes in the system can be reached within a maximum of six intermediate movements, reflecting a relatively compact and well-connected network structure. A smaller network diameter indicates that nodes can be connected through relatively short paths within the movement network, suggesting a greater degree of structural connectivity and potentially facilitating dissemination across the network if an infectious agent is introduced. However, network topology alone cannot predict the speed or magnitude of HPAI spread. Epidemic dynamics are additionally influenced by factors such as transmission probability, movement frequency and timing, duration of infectiousness, prevalence, biosecurity measures, and other epidemiological characteristics. Therefore, the network metrics identified in this study should be interpreted as indicators of potential dissemination pathways and structural connectivity, rather than direct predictors of epidemic speed or transmission dynamics.

In a comparative context, this value is notably low relative to the diameter values reported by Lezaar et al. (2024) [32] for the national Moroccan sheep movement network, where diameters of 12 and 13 were recorded during the festive and regular periods, respectively. In that network, pathogens would need to traverse a considerably longer chain of connections before reaching the most peripheral municipalities, indicating a naturally slower spread dynamic compared to what our findings suggest for the poultry sector. The markedly reduced diameter observed in the Casablanca-Settat poultry network can be attributed to two combined factors: the relatively small size of the network (126 municipalities) and the high concentration of transport flows within a geographically constrained area, both of which contribute to shortening the average distance between nodes.

This structural observation is consistent with the results of Kurscheid et al. (2017) [31], who demonstrated in their study of live bird market networks in Indonesia that smaller network diameters were consistently associated with faster and more efficient viral propagation between nodes. Collectively, these results suggest that the structural compactness of the Casablanca-Settat poultry network constitutes a significant epidemiological vulnerability, warranting heightened surveillance, particularly at nodes occupying central positions along the shortest paths connecting distant municipalities.

Spatial mapping of the network further revealed that poultry movement flows extend well beyond the boundaries of the study area. The network expands into two directly adjacent regions, Rabat-Salé-Kénitra and Marrakech-Safi, where a substantial concentration of movements was recorded. Moreover, flows were observed reaching geographically distant regions, including Tanger-Tétouan-Al Hoceïma, Fès-Meknès, and Souss-Massa, reflecting a connectivity pattern that transcends the regional scale and underscores the potential for inter-regional disease dissemination.

This spatial structure carries significant epidemiological implications. In the event of an HPAI outbreak, the virus would not remain confined to the Casablanca-Settat region but could rapidly extend along this inter-regional transport movement, establishing new infection in areas far removed from the initial source. Guinat et al. (2022), in their phylodynamic study of HPAI spread across Europe, demonstrated that long-distance links between poultry production areas represented one of the primary drivers of viral dissemination, making epidemic management particularly complex when the network extends beyond local administrative boundaries [5].

This observation is directly applicable to the Moroccan context. The network movement in this study, linking the Casablanca-Settat region to distant parts of the country, suggests that a virus introduced into this network could spread rapidly and prove difficult to contain at a national scale. This highlights the necessity for inter-regional coordination in HPAI surveillance and control efforts across Morocco and not just within the boundaries of individual administrative regions.

Centrality parameters allow for the identification of critical points of vulnerability and potential HPAI dissemination within the network. The five municipalities with the highest total degree, in descending order, were: Had Soualem, the Hay Mohammadi, Sahel Oulad H’Riz, Labrikiyine, and Settat. These municipalities concentrate the largest number of connections in the network and play a determining role in the dynamics of HPAI spread. Had Soualem, Labrikiyine, Sahel Oulad H’Riz, Cherrat, and Hay Mohammadi recorded the highest out-degree values. Several authors have proposed that out-degree is a key indicator for identifying nodes most likely to disseminate infections, and have suggested that risk-based surveillance programs should prioritize these nodes [28]. This finding is consistent with the results of Kurscheid et al. (2017), who identified high out-degree nodes in Indonesian poultry networks as the primary candidates for disease control programs, including enhanced surveillance, improved biosecurity measures, and HPAI awareness campaigns [31].

Hay Mohammadi in Casablanca recorded the highest in-degree value in the network, owing to the presence of the two largest wholesale markets for poultry and table eggs in Morocco. The municipalities of Had Soualem, Sahel Oulad H’Riz, Settat, and Sidi Bou Othamane followed. Notably, Had Soualem, Sahel Oulad H’Riz, and Hay Mohammadi exhibited simultaneously high in-degree and out-degree values, conferring upon them a dual role as both major sources and major destinations of transport flows, what Kurscheid et al. (2017) describe as “super-spreader” nodes, carrying the highest potential for disseminating infection throughout the entire network [31]. This configuration reflects the fact that Had Soualem and Sahel Oulad H’Riz alone account for more than 40% of the poultry feed mills and 50% of the industrial slaughterhouses present in the network, while Hay Mohammadi serves as the principal hub for the reception and redistribution of live poultry and table eggs at the national level.

By contrast, certain municipalities displayed high in-degree but low out-degree values, such as Settat (in-degree: 19, out-degree: 2) and Sidi Bou Othamane (in-degree: 12, out-degree: 1), rendering them highly vulnerable to contamination from other nodes while contributing little to onward dissemination. Conversely, municipalities such as Labrikiyine (out-degree: 21, in-degree: 0), Cherrat (out-degree: 19, in-degree: 1), and Tit Mellil (out-degree: 16, in-degree: 0) function primarily as sources of viral spread toward other municipalities. These three distinct node categories play complementary yet differentiated roles in HPAI transmission dynamics, which argues for a surveillance strategy that is tailored to the specific centrality profile of each municipality rather than applying a uniform approach across the network.

Regarding betweenness centrality, the municipalities recording the highest values, in descending order, were: Had Soualem, the Hay Mohammadi district, Sahel Oulad H’Riz, Cherrat, and Settat. These municipalities connect a large number of other nodes within the network and serve as key transit points for movement flows. In the event of an outbreak, restricting movements originating from these municipalities could prove instrumental in containing disease spread across the network. This result is consistent with the findings of Mulatti et al. (2018) [6], who, in their analysis of the HPAI H5N8 epizootic in Italy (2016–2017), demonstrated that high-betweenness nodes played a critical role in viral dissemination across geographically distinct areas, and that prioritizing these nodes in control strategies significantly reduced the overall scale of outbreaks. When combining all three centrality indicators (out-degree, in-degree, and betweenness) the three most influential municipalities in the network, in descending order, were Had Soualem, Hay Mohammadi, and Sahel Oulad H’Riz.

Network analysis identified a strong component including 57 municipalities strongly linked through poultry transport movements, spanning 11 provinces across three regions of the kingdom of Morocco: Casablanca-Settat, Rabat-Salé-Kénitra, and Marrakech-Safi. This means that a directed path exists between every pair of municipalities within it. Structurally, this indicates that no municipality in this component is isolated from the others by the direction of trade flows. It should be emphasized that this describes theoretical reachability through existing transport routes, not an expectation that an introduction at any point would propagate throughout the component: whether such propagation occurred would depend on transmission probability along each movement, on the biosecurity measures applied, and on the timing of movements relative to the infectious period.

This finding is consistent with the results reported by Lezaar et al. (2024) [32] for the Moroccan sheep movement network, in which the strong component (described by the authors as a “predictor of the final size of epizootics”) was found to determine the maximum potential extent of disease spread. By analogy, rapid and targeted intervention within the strong component of the Casablanca-Settat poultry network would be critical to limiting the scale of any future HPAI epidemic. Along the same lines, Chaters et al. (2019) [33], in their analysis of livestock movement networks in East Africa, demonstrated that even partial movement data were sufficient to identify the strong component and the most critical nodes, making a convincing case for the routine collection of movement data in emerging economies. This recommendation applies directly to the Moroccan context.

Six municipalities were identified as cutpoints: Hay Mohammadi, Had Soualem, Sahel Oulad H’Riz, Settat, Skhour Rhamna, and Sidi Bou Othmane. Removing these nodes from the network would substantially reduce its overall connectivity and, consequently, lower the risk of HPAI dissemination across the region. This result is consistent with the findings of Lezaar et al. (2024) [32], who identified 63 strategic cutpoint municipalities during the regular period and 68 during the festive period in the Moroccan sheep movement network, and with those of Salathé and Jones (2010) [34], who demonstrated that in networks comprising multiple communities, intervention strategies targeting nodes that bridge different communities are considerably more effective than those focusing solely on the most highly connected nodes. The jackknife stability analysis further supports the reliability of the hub and cutpoint findings reported here: the leading hub municipalities identified in this study were consistently retained across all leave-one-node-out iterations, suggesting that the prioritization of these locations for HPAI surveillance reflects a genuine structural property of the transport network rather than an artifact of the specific sample of municipalities surveyed.

Incorporating movement frequency, where reliably measurable, represents a valuable direction for future work, allowing comparison between topological importance and volume-weighted transmission risk.

Resilience analysis of the network showed that the sequential removal of nodes based on betweenness centrality led to a rapid reduction in the size of the largest connected component, confirming that these nodes are essential to maintaining the overall connectivity of the network. Node removal based on in-degree and out-degree also had a significant impact on network connectivity, though these approaches proved slightly less effective than betweenness-based removal in fragmenting the network. Costa et al. (2025), analyzing the vulnerability of the cattle movement network of Minas Gerais, Brazil, likewise identified betweenness and out-degree as the most effective measures for guiding targeted intervention [35], corroborating the ordering obtained here.

By contrast, random node removal resulted in a slow and gradual degradation of connectivity, illustrating the inherent robustness of heterogeneous networks against non-targeted disruptions. This finding is in line with the observations of both Kurscheid et al. (2017) [31] and Napp et al. (2018) [7], who independently highlighted that scale-free poultry networks are largely resistant to random control measures while remaining highly vulnerable to targeted strategies focused on hub nodes. Sequeira et al. (2025) [36], in their systematic review of 203 studies, further confirmed that resilience analysis represents one of the most relevant applications of network analysis in veterinary epidemiology, particularly for optimizing the allocation of limited resources in middle-income countries.

Taken together, these findings delineate a concrete basis for prioritizing HPAI surveillance in the poultry sector of the Casablanca-Settat region. The network is markedly heterogeneous: a small number of municipalities concentrate a disproportionate share of connections, a structure that none of the 100 random networks of comparable order and size reproduced. Three municipalities—Had Soualem, Hay Mohammadi and Sahel Oulad H’Riz—combine high in-degree, high out-degree and high betweenness, placing them at the structural core of the regional transport system, while six further municipalities act as cutpoints whose removal would disconnect otherwise linked parts of the network.

The resilience simulation makes the practical consequence of this architecture explicit: removing nodes in decreasing order of centrality fragments the network considerably faster than removing them at random, which is to say that surveillance and control effort concentrated on a small number of locations would reduce connectivity far more than the same effort distributed uniformly across the region. The leave-one-node-out analysis further indicates that this ranking reflects a stable property of the transport network rather than an artifact of the particular municipalities surveyed. Where surveillance resources are limited, these locations therefore constitute rational points of entry for risk-based sampling, for the targeting of biosecurity audits at collection and marketing points, and for the prioritization of movement controls in the event of an incursion.

### Study Limitations

The main limitation is the absence of temporal data. Movements were aggregated into a static network because their dates could not be reliably ascertained through the questionnaire survey, and because the objective was to characterize connectivity structure rather than the chronological sequence of contacts. The measures reported therefore describe the routes potentially available for dissemination rather than realized transmission pathways. For the same reason, the two survey periods were pooled rather than tested for period effects; should period-specific drivers of mobility exist, the rankings reported here would represent their average.

The network was also unweighted, since movement volumes could no more be reliably ascertained than their dates. Connections carrying very different numbers of birds were thus assigned equal importance, and the central nodes identified reflect connectivity patterns rather than movement intensity. Weighted and multilayer formulations provide an established methodological route for incorporating contact intensity and multiple contact types into such analyses [37], and represent the natural extension of this work once movement volumes can be verified.

Movements were declared in free-text form and not cross-checked against traceability records; as the commodities concerned likely differ in transport regularity and recall accuracy, connections carrying less regular flows may be under-represented.

More broadly, the network characterizes the structural opportunity for dissemination arising from vehicle movements, not the holding-level determinants that condition whether a contact results in transmission—species and production type, holding size, vehicle sharing, cleaning and disinfection. Documenting these across the region’s approximately 2500 poultry units would constitute a distinct investigation. Linking the present characterization with such determinants, ideally through sanitary authority records, would allow topological importance to be weighted by holding susceptibility. The ranking proposed here is accordingly a structural prioritization, to be integrated with these factors in a fuller risk assessment rather than substituted for one.

Finally, extending this analysis to other Moroccan regions would identify further vulnerable areas and support an evidence-based national strategy for HPAI control.

## 5. Conclusions

This study was conducted as a pilot investigation limited to the Casablanca-Settat region of Morocco and was not extended to the national territory, owing to the practical difficulty of collecting poultry movement data at a country-wide scale. The findings presented here should therefore be interpreted as region-specific to the sampled municipalities, with extension to other regions and ultimately to the national scale representing an important direction for future work, contingent on the development of more systematic and verifiable data-collection mechanisms.

Through the combined use of three centrality measures—out-degree, in-degree, and betweenness centrality—Had Soualem, the Hay Mohammadi district, and Sahel Oulad H’Riz emerged as the most strategically significant nodes. Acting simultaneously as major sources of dissemination, key destinations for poultry flows, and critical bridges between distinct parts of the network, these communes occupy a central position in any HPAI prevention and response framework.

The network’s strong component, encompassing 57 communes across 11 provinces and three regions, delineates the maximum potential zone of disease diffusion in the event of an HPAI introduction. Equally important, the identification of six cutpoints—Hay Mohammadi, Had Soualem, Sahel Oulad H’Riz, Settat, Skhour Rhamna, and Sidi Bou Othmane—offers actionable intervention leverage: targeted movement restrictions at these nodes would substantially fragment the network and curb the risk of large-scale pathogen spread.

These findings call for the implementation of risk-based surveillance and control strategies centered on the identified critical nodes, supported by real-time movement tracking systems, modernization of poultry trade infrastructure, and strengthened awareness among sector stakeholders of the epidemiological risks inherent to the transport of live poultry and poultry products.

## Figures and Tables

**Table 1:** Number of sampled municipalities and sampling rate for each stratum.

Number of Poultry Farms per Municipality	Municipalities in the Region	Sampling Rate	Municipalities Sampled
50–107 (very important)	8	50%	4
25–50 (important)	22	25%	6
5–25 (less important)	68	25%	17
1–5 (much less important)	40	25%	10
Total	138	—	37

**Table 2:** Global descriptive parameters of the transport network.

Betweeness	Total Degree	In-Degree	Out-Degree
**Had Soualem (5863.62)**	Had Soualem (95)	Hay Mohammadi (46)	Had Soualem (58)
**Hay Mohammadi (3601.05)**	Hay Mohammadi (63)	Had Soualem (37)	Labrikiyne (21)
**Sahel Oulad H’Riz (725.67)**	Sahel Oulad H’Riz (40)	Sahel Oulad H’Riz (20)	Sahel Oulad H’Riz (20)
**Cherrat (653.63)**	Labrikiyne (21)	Settat (19)	Cherrat (19)
**Settat (390.71)**	Settat (21)	Sidi Bou-Othmane (12)	Hay Mohammadi (17)
**M. Abdellah Amghar (355.18)**	Cherrat (20)	Dar Chaffai (8)	Tit Mellil (16)
**Oulad Frej (308.48)**	Chtouka (18)	Guisser (8)	M. Abdellah Amghar (14)
**Skhirat (270.75)**	M. Abdellah Amghar (17)	Fdalate (7)	Skhour Rhamna (14)
**Skhour Rhamna (267.05)**	TitMellil (16)	Sidi Hajjaj Oued Hassar (7)	Chtouka (12)
**Sidi Bou-Othmane (235.44)**	Skhour Rhamna (15)	ElMansouria (7)	Tiddas (10)
**Bir Jdid (211.24)**	Dar Chaffai (13)	Chtouka (6)	Skhirat (8)
**Benslimane (143.25)**	Sidi Bou-Othmane (13)	Sidi El Mekki (6)	Mzilate (8)
**Dar Chaffai (138.52)**	Bir Jdid (11)	Ain Dorbane (6)	Bir Jdid (7)
**Fdalate (132.36)**	Guisser (11)	Mrizigue (6)	Jamaat Shaim (6)
**Sidi Moussa Ben-Ali (125.38)**	Fdalate (10)	Sidi MoussaBenAli (6)	Dar Chaffai (5)

**Table 3:** Comparison of the observed centralization indices with 100 Erdős–Rényi random networks of identical order and size.

Centralization Indices	Observed	Random Networks, Mean ± SD (*n* = 100)	*p*
In-degree	0.358	0.039 ± 0.007	<0.001
Out-degree	0.437	0.042 ± 0.008	<0.001
Betweenness	0.0029	0.0006 ± 0.0001	<0.001

**Table 4:** Ranking of the 15 leading municipalities according to their centrality parameters.

**Number of nodes**	126
**Number of links**	376
**Density**	0.023
**Reciprocity**	0.259
**Diameter**	6
**Heterogeneity coefficient (Kappa) for in-degree**	4.86
**Heterogeneity coefficient (Kappa) for out-degree**	5.74
**Heterogeneity coefficient (Kappa) for total degree**	4.47

## Data Availability

The original contributions presented in this study are included in the article. Further inquiries can be directed to the corresponding author.

## Abbreviations

The following abbreviations are used in this manuscript:

HPAI	Highly Pathogenic Avian Influenza
SNA	Social Network Analysis
ONSSA	National Office for Food Safety
QGIS	Quantum GIS

## Appendix A

**Table A1:** Definition of network analysis indicators and their epidemiological implications [38].

Type of Measure	Concept	Indicator	Definition and Epidemiological Implication
Global	Network—level cohesion	Density	A value ranging from 0 to 1 that provides information on the overall connectivity of the network based on the proportion of links established between nodes. It provides an indication of the potential speed at which an epidemic may spread within the network.
		Diameter	The diameter of a graph is the greatest distance between two nodes in the network. This measure, like the average distance, provides information about how easily nodes within the network can interact or communicate.
		Reciprocity	Reciprocity corresponds to strong connectivity in graph theory. A graph is strongly connected if, for every pair of distinct nodes, there exists a path from one node to the other in both directions. This indicator highlights the importance of bidirectional contacts for disease transmission between nodes.
Local	Incoming activity centrality	In-degree	Indicates the number of incoming movements and reflects the susceptibility of a node to infection.
	Outgoing activity centrality	Out-degree	Indicates the number of outgoing movements and reflects the potential infectious influence of a node on other connected nodes.
	Betweenness centrality	Betweenness	Measures the number of times a node lies on the shortest path between other pairs of nodes. It reflects the ability of a node to act as a bridge controlling flows within the network and highlights its role as a key mobility hub.
Detection of cohesive subgroups	Cohesion	Component	A component is the largest set of connected nodes within the network. A weak component occurs when nodes are connected but not all nodes can reach each other through directed paths. In epidemiological terms, this may correspond to outbreaks that do not affect the entire network.
		Strong Components	Strong components are often studied as predictors of the final size of epizootics. They represent subsets of nodes that are strongly connected, meaning that each node can reach all other nodes in the subset. These structures may facilitate rapid disease dissemination among highly connected establishments.
Articulation points	Cohesion and fragmentation	Cutpoints	Cutpoints are critical nodes whose removal increases the number of network components and the level of fragmentation. When such nodes also have high centrality, the network becomes highly vulnerable. These nodes are important targets for disease control strategies aimed at preventing epidemic spread.
